# PredAOT: a computational framework for prediction of acute oral toxicity based on multiple random forest models

**DOI:** 10.1186/s12859-023-05176-5

**Published:** 2023-02-24

**Authors:** Jae Yong Ryu, Woo Dae Jang, Jidon Jang, Kwang-Seok Oh

**Affiliations:** 1 Department of Biotechnology, Duksung Women’s University, 33 Samyang-Ro 144-Gil, Dobong-gu, Seoul, 01369 Republic of Korea; 2 Center for Research and Development, Oncocross Ltd., Seoul, Republic of Korea; 3 Data Convergence Drug Research Center, Korea Research Institute of Chemical Technology, 141, Gajeong-ro, Yuseong-gu, Daejeon, 34114 Republic of Korea; 4 Department of Medicinal and Pharmaceutical Chemistry, University of Science and Technology, 176 Gajeong-Ro, Yuseong-gu, Daejeon, 34129 Republic of Korea

**Keywords:** Acute oral toxicity, Machine learning, Random forest, Drug discovery

## Abstract

**Background:**

Acute oral toxicity of drug candidates can lead to drug development failure; thus, predicting the acute oral toxicity of small compounds is important for successful drug development. However, evaluation of the acute oral toxicity of small compounds considered in the early stages of drug discovery is limited because of cost and time. Here, we developed a computational framework, PredAOT, that predicts the acute oral toxicity of small compounds in mice and rats.

**Methods:**

PredAOT is based on multiple random forest models for the accurate prediction of acute oral toxicity. A total of 6226 and 6238 compounds evaluated in mice and rats, respectively, were used to train the models.

**Results:**

PredAOT has the advantage of predicting acute oral toxicity in mice and rats simultaneously, and its prediction performance is similar to or better than that of existing tools.

**Conclusion:**

PredAOT will be a useful tool for the quick and accurate prediction of the acute oral toxicity of small compounds in mice and rats during drug development.

## Background

After evaluating the effectiveness of drugs in the process of discovery and development of new drugs, preclinical tests were performed to evaluate their toxicity in animals prior to clinical trials [1]. Preclinical toxicity testing can predict toxic responses in humans, determine safe doses in clinical trials, and monitor toxicity-related symptoms and target organs in patients [2]. After having confirmed the safety of drugs by evaluating various toxicities, such as acute toxicity, nephrotoxicity, cardiotoxicity, reproductive toxicity, and genotoxicity, clinical trials can be performed.

Acute toxicity is defined as deleterious toxicological effects of a chemical from single or multiple exposures over a short duration (usually < 24 h) [3]. Studies on acute toxicity have examined various routes of exposure (e.g., oral, dermal, and inhalation) using rodents, such as mice and rats, to assess lethal doses. During the drug development process, acute toxicity is generally evaluated using acute oral toxicity (AOT) tests that assess acute toxic reactions and the lethal dose 50 (LD_50_) after a single oral administration to rodents [4, 5]. The LD_50_ is defined as the dose of the test substance that can kill 50% of animals within 24 h of exposure.

AOT tests in animals are conducted after confirming the efficacy of a drug [5]. Therefore, if drug development fails owing to AOT at this stage, economic loss of development costs incurs. However, as dozens to thousands of compounds are considered drug candidates in the early stages of development, performing AOT tests on all compounds is limited by time and cost issues. Therefore, it is important to evaluate the AOT of such compounds in the early stages of drug development.

To address these issues, various prediction models have been developed based on the results of AOT tests of thousands of compounds [6–8]. In addition, various software programs that predict the AOT of small compounds based on machine learning models are currently available [7–10]. However, several aspects need to be improved for the development of an AOT prediction model. First, it is necessary to develop a computational framework that can predict the AOT in both mice and rats. AOT evaluation of small compounds is still being performed using either mice or rats. Second, it is necessary to further improve the prediction performance of models. One strategy to improve the performance of a model is to build it so that it can properly consider the distribution of the data. For example, using data composed of skewed LD_50_ values for model training may reduce prediction performance [11].

In the present study, we propose a new computational framework, PredAOT, that predicts the AOT for a given compound in mice and rats. PredAOT is based on multiple random forest models for AOT prediction. For the development of PredAOT, we used a total of 6,226 and 6,238 compounds whose AOT was evaluated in mice and rats, respectively. Moreover, we compared the prediction performance of PredAOT with that of other existing tools. PredAOT is a useful tool for predicting the acute oral toxicity of small compounds during drug development.

## Results and discussion

### Development of the PredAOT framework

To develop a computational framework (i.e., PredAOT) for accurate prediction of AOT, we first collected data on the AOT of compounds (i.e., LD_50_) reported for mice and rats. The AOT data for mice for 6226 compounds were obtained from the OCHEM database [7]. The AOT data for rats for 6238 compounds were obtained from the literature [6].

According to the GHS Classification, the AOT level of compounds can be divided into five categories [12] (Table 1). For example, compounds in Category 1 are toxic, while Category 5 compounds are less likely to be toxic. Additionally, we found that the distribution of LD_50_ values for the compounds was skewed towards categories 4 and 5 in both mice and rats. This data imbalance can adversely affect model training, such as overfitting. To address this issue, we decided to classify the AOT of compounds into two categories (i.e., “toxic” and “less or non-toxic”) instead of five categories (Table 2); compounds with LD_50_ ≤ 300 mg/kg and compounds with LD_50_ > 300 mg/kg. Notably, although a compound is classified as less or non-toxic, it does not necessarily have to involve no AOT at all. During the model development, LD_50_ values were transformed to log10 transformed LD_50_ values.

**Table 1:** Number of compounds in datasets for each acute oral toxicity category

Toxicity category	LD_50_ (mg/kg)	Hazard statement	Mouse	Rat
Category 1	< 5	Fatal	67	173
Category 2	5–50	Fatal	282	490
Category 3	50–300	Toxic	1183	1103
Category 4	300–2000	Harmful	3413	2560
Category 5	2000–5000	May be harmful	1281	1912

**Table 2:** Number of compounds in the “less or non-toxic” and “toxic” datasets

	Less or non-toxic	Toxic
Mouse	4,694	1,532
Rat	4,472	1,766

**Fig. 1:**
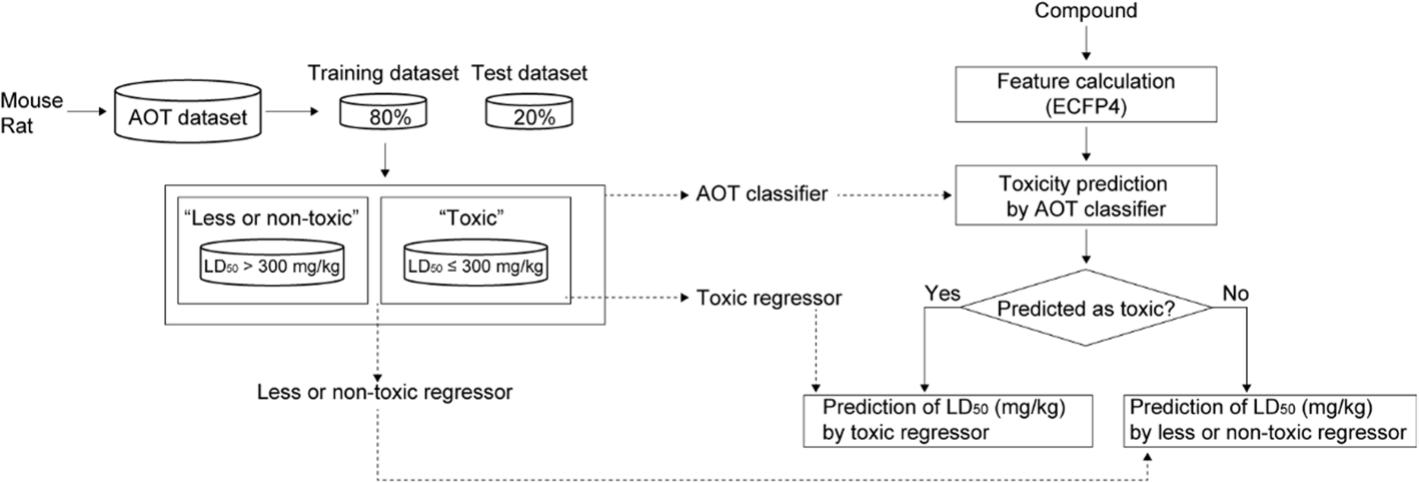
Overall scheme of PredAOT. PredAOT uses the chemical structure as an input. Thereafter, the molecular fingerprint (i.e., ECFP4) is used as an input feature for models in PredAOT. PredAOT is composed of one classification model (i.e., “AOT classifier”) and two regression models (i.e., “toxic regressor” and “less or non-toxic regressor”). The AOT classifier is used for prediction as “toxic” (LD_50_ ≤ 300 mg/kg) or “less or non-toxic” (LD_50_ > 300 mg/kg) for a given compound. If a compound is predicted to be toxic, PredAOT predicts the LD_50_ of the compound using the toxic regressor trained with compounds with LD_50_ ≤ 300 mg/kg. If a compound is predicted to be less or non-toxic, PredAOT predicts the LD_50_ of the compound using the less or non-toxic regressor trained with compounds with LD_50_ > 300 mg/kg. All these procedures are equally applied to the AOT prediction process in mice and rats

We then used the datasets to train a binary classification model, called “AOT classifier,” that predicts AOT as toxic (i.e., LD_50_ ≤ 300 mg/kg) or less or non-toxic (i.e., LD_50_ > 300 mg/kg) for a given compound. In addition, as shown in Fig. 1, toxic and less or non-toxic datasets were used to train two regression models called “toxic regressor” and “less or non-toxic regressor”, respectively. In particular, the “toxic regressor” was trained with the toxic dataset (i.e., LD_50_ ≤ 300 mg/kg) and the “less or non-toxic regressor” was trained with the less or non-toxic dataset (i.e., LD_50_ > 300 mg/kg).

To summarize the AOT prediction process in the PredAOT, “AOT classifier” first predicts AOT as “toxic” or “less or non-toxic” for a given compound. Thereafter, if a compound is predicted as toxic, the “toxic regressor” predicts the LD_50_ of the compound; otherwise, the “less or non-toxic regressor” predicts the LD_50_ of the compound. All procedures were equally applied to the AOT prediction process in mice and rats.

### Optimization and evaluation of AOT prediction models

To build an optimal AOT prediction model, we constructed and evaluated six different machine learning models: a message passing neural network (MPNN) based on graph neural networks, MPNN with molecular fingerprints, MPNN with molecular descriptors, random forest (RF), support vector machine (SVM), and artificial neural network (ANN) models based on molecular fingerprints (see Materials and Methods). The training dataset was used for model training to determine the model with the best prediction performance. A test dataset was used to assess the performance of the final model.

**Fig. 2:**
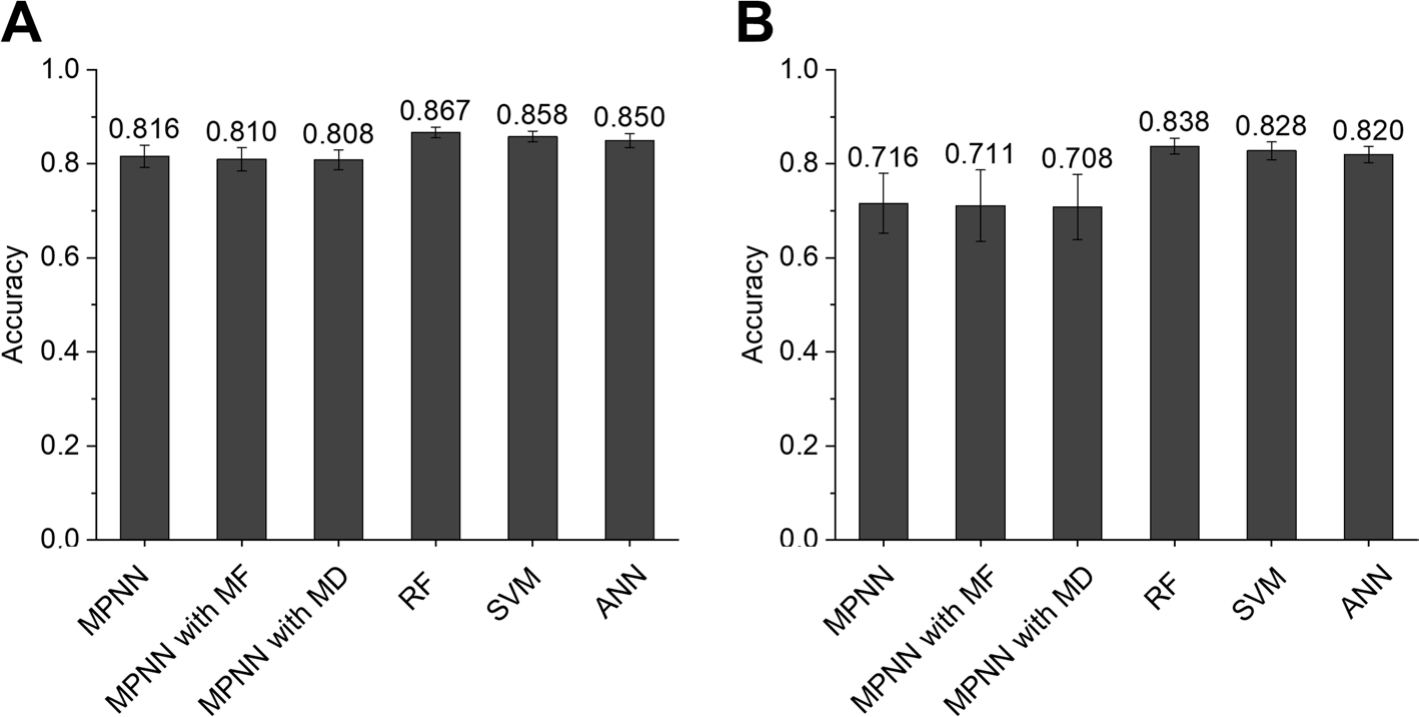
Cross-validation prediction performances of the MPNN, MPNN with MF, MPNN with MD, RF, SVM, and ANN models for the AOT classifier using mouse (**A**) and rat (**B**) datasets. Each performance metric value was calculated by five-fold cross-validation. MPNN, message passing neural network; MF, molecular fingerprint; MD, molecular descriptor; RF, random forest; SVM, support vector machine; ANN, artificial neural network

**Fig. 3:**
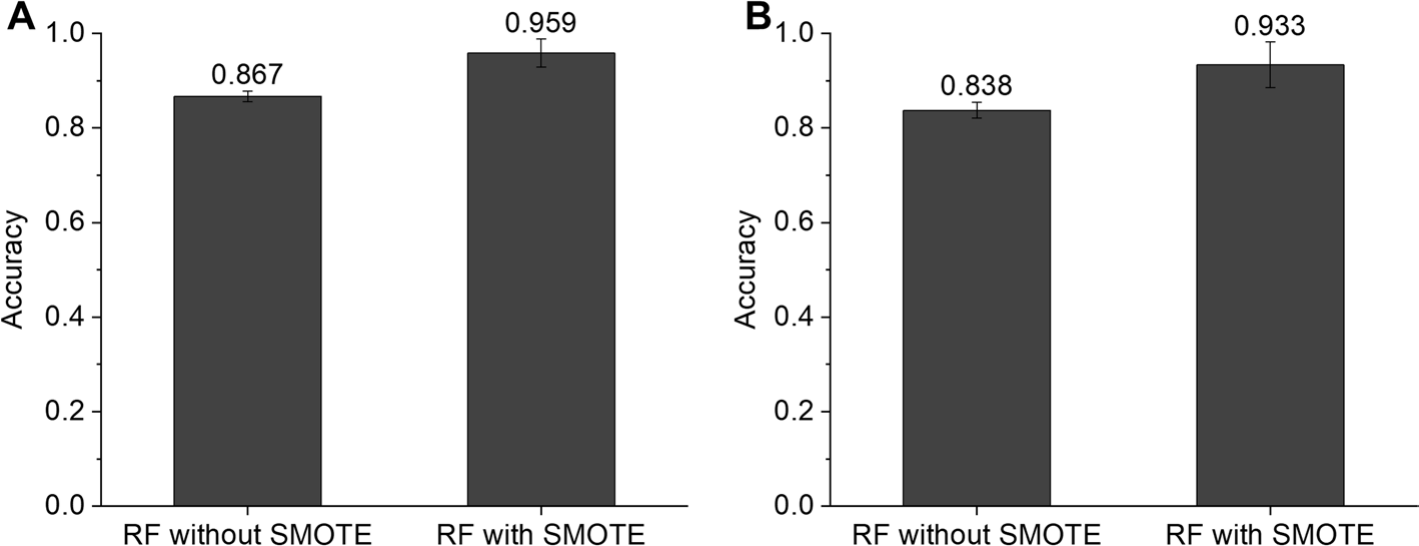
Cross-validation prediction performances of RF models with and without SMOTE using mouse (**A**) and rat (**B**) datasets. MPNN, message passing neural network; RF, random forest; SVM, support vector machine; ANN, artificial neural network

First, we optimized the binary classification model (i.e., “AOT classifier”) used to predict the AOT of a given compound in mice and rats. To this end, we evaluated various hyperparameters using a grid search technique with fivefold cross-validation, and used accuracy as the model performance metric. RF showed the highest accuracy for mice (0.8672) and rats (0.8377; Fig. 2). To further improve the prediction performance of the RF model, we applied an oversampling approach to manage imbalanced data (Table 2). Specifically, we used the synthetic minority oversampling technique (SMOTE), which is a popular algorithm used to generate artificial data [13]. In doing so, RF with SMOTE showed better prediction performance than RF without SMOTE: accuracies of 0.9586 and 0.9335 in mice and rats, respectively (Fig. 3). In addition, we evaluated the prediction performance using the test dataset. The RF model with SMOTE showed an area under the receiver operating characteristic (AUROC) of 0.7778, Matthew’s correlation coefficient (MCC) of 0.5514, positive predictive value (PPV) of 0.6627, and negative predictive value (NPV) of 0.8845 in mice, and an AUROC of 0.7442, MCC of 0.4929, PPV of 0.6435, and NPV of 0.8539 in rats (Table 3). Based on these results, we used RF models with SMOTE in both mice and rats in the PredAOT framework.

**Table 3:** Prediction performance of classification models in PredAOT on the test dataset

	AUROC	MCC	PPV	NPV
Mouse	0.7778	0.5514	0.6627	0.8845
Rat	0.7442	0.4929	0.6435	0.8539

AUROC, area under receiver operating characteristic; MCC, Matthew’s correlation coefficient; PPV, positive predictive value; NPV, negative predictive value

**Fig. 4:**
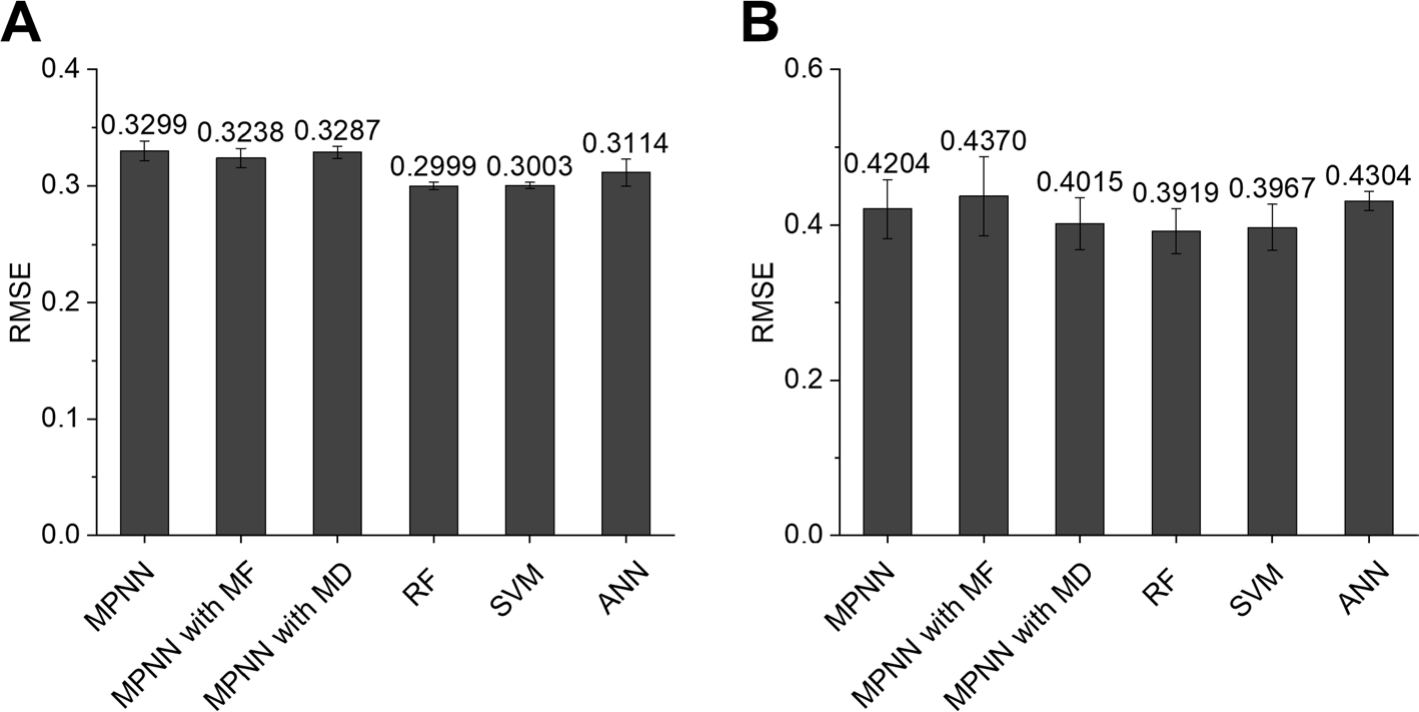
Cross-validation prediction performances of the MPNN, MPNN with MF, MPNN with MD, RF, SVM, and ANN models for toxic regressors using mouse (**A**) and rat (**B**) toxic datasets (i.e., LD_50_ ≤ 300 mg/kg). MPNN, message passing neural network; MF, molecular fingerprint; MD, molecular descriptor; RF, random forest; SVM, support vector machine; ANN, artificial neural network

**Fig. 5:**
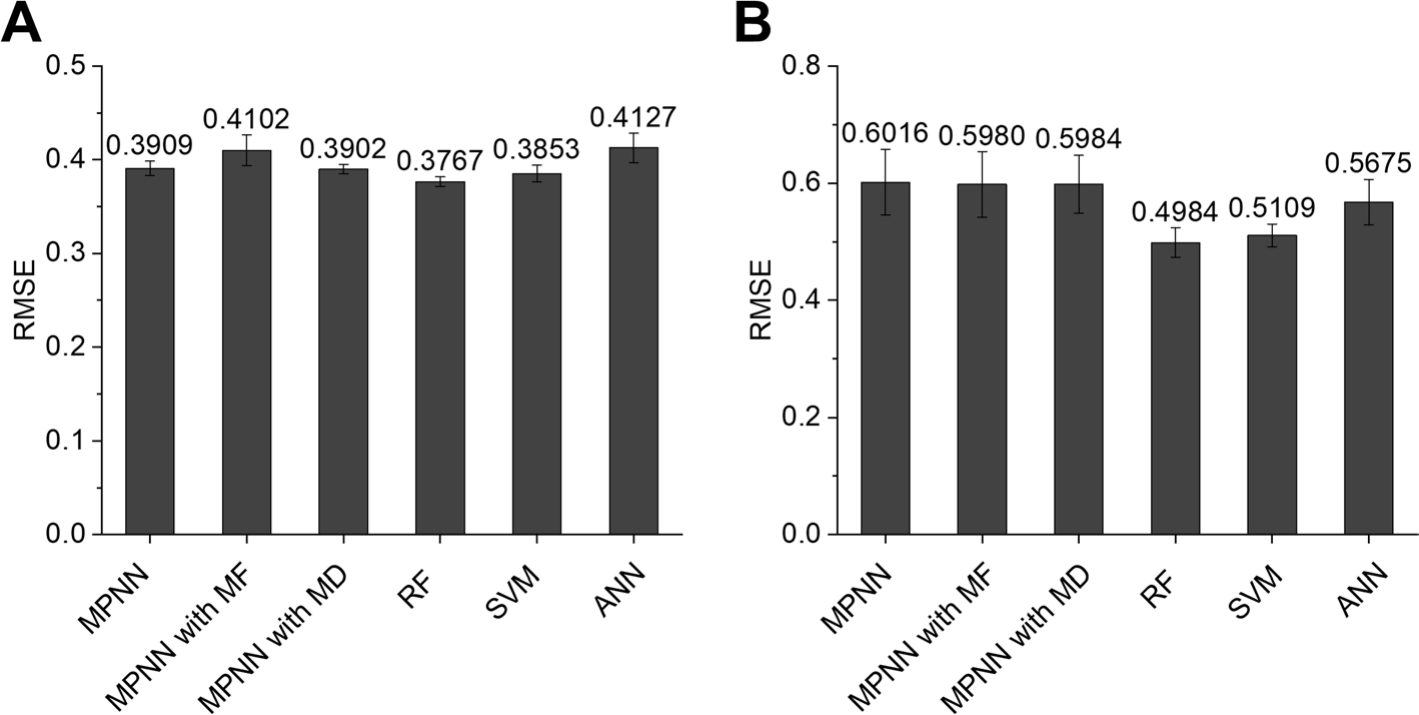
Cross-validation prediction performances of the MPNN, MPNN with MF, MPNN with MD, RF, SVM and ANN models for the non-toxic regressor using mouse (**A**) and rat (**B**) less or non-toxic datasets (i.e., LD_50_ > 300 mg/kg). MPNN, message passing neural network; MF, molecular fingerprint; MD, molecular descriptor; RF, random forest; SVM, support vector machine; ANN, artificial neural network

As shown in Fig. 1, the AOT of the compound was first predicted to be “toxic” or “less or non-toxic” using an AOT classifier in mice and rats. The LD_50_ value (mg/kg) was subsequently quantitatively predicted using one of the regression models (i.e., “toxic regressor” or “less or non-toxic regressor”) according to the prediction result of the AOT classifier. Here, we optimized both regressors (i.e., “toxic regressor” or “less or non-toxic regressor”). The prediction performance of these regressors was evaluated through five-fold cross-validation using the training dataset. The root-mean-square error (RMSE) was used as the performance metric for the regression models. Consequently, RF showed the lowest RMSE, i.e., the best performance, in the "toxic regressor” and “less or non-toxic regressor” in both mice and rats. The RF model for both regressors showed an RMSE of 0.2999 and 0.3767 in mice, respectively (Figs. 4A, 5A) and 0.3919 and 0.4984 in rats, respectively (Figs. 4B, 5B). Thereafter, we evaluated the prediction performance using the test dataset. In mice, the toxic regressor showed an RMSE of 0.3806 and an R^2^ of 0.3557 on the test dataset (Table 4), whereas the less or non-toxic regressor showed an RMSE of 0.2923 and an R^2^ of 0.3881. In rats, the toxic regressor showed an RMSE of 0.5323 and an R^2^ of 0.3065 on the test dataset, whereas the less or non-toxic regressor showed an RMSE of 0.3863 and an R^2^ of 0.2702.

**Table 4:** Prediction performance of regression models in PredAOT on the test dataset**.** RMSE and R^2^ values are calculated using log10 transformed LD50 values

	Regression model	RMSE	R^2^
Mouse	Less or non-toxic	0.2923	0.3881
	Toxic	0.3806	0.3557
Rat	Less or non-toxic	0.3863	0.2702
	Toxic	0.5323	0.3065

RMSE, root mean squared error; R^2^, R-squared

### Prediction performance of PredAOT compared with other prediction models

We compared the prediction performance of PredAOT with that of other existing tools. First, we compared the prediction performance of PredAOT in mice. Thereafter, we compared the prediction performance of PredAOT with OCHEM Predictor using our test dataset [7]. The OCHEM Predictor predicted the LD_50_ (mg/kg) result in the same manner as PredAOT, such that predicted values could be directly compared. We compared the prediction performance for each toxicity group in Table 1. PredAOT yields relatively low RMSE values (i.e., improved performance) in groups with relatively little training data (e.g., Categories 1 and 2) (Table 5). Notably, the performance comparison indicates that the test dataset may have been used as training data in OCHEM Predictor.

**Table 5:** Comparison of RMSE values predicted by PredAOT with OCHEM Predictor on the mouse test dataset

Toxicity category	OCHEM Predictor	PredAOT
Category 1	193.11	82.13
Category 2	273.24	294.64
Category 3	805.42	289.55
Category 4	513.90	533.00
Category 5	1645.57	1645.25

RMSE, root mean squared error

Second, we compared the prediction performance of PredAOT in rats with that of BESTox and aiQSAR using our test dataset [9, 10]. The prediction results of BESTox and aiQSAR were not directly comparable with the prediction results of PredAOT ​​because the unit for LD_50_ was not mg/kg. Therefore, we compared the prediction results using two correlation coefficients: Pearson’s correlation coefficient (Pearson’s r) and Spearman's rank correlation coefficient (Spearman's r). PredAOT showed the highest Pearson’s r of 0.7984 and Spearman's r of 0.7340 compared to BESTox and aiQSAR (Table 6).

**Table 6:** Comparative performance evaluation of PredAOT with BESTox and aiQSAR on the rat test dataset

	Pearson's r	Spearman's r
BESTox	0.7170	0.6978
aiQSAR	− 0.8989	− 0.8918
PredAOT	0.7984	0.7639

Although we did not compare LD_50_ values directly, PredAOT performed comparably or better than existing tools. PredAOT does not outperform other existing tools; however, it can be a useful tool for predicting AOT. In addition, it has the advantage of being able to predict AOT in mice and rats simultaneously.

## Conclusions

**Fig. 6:**
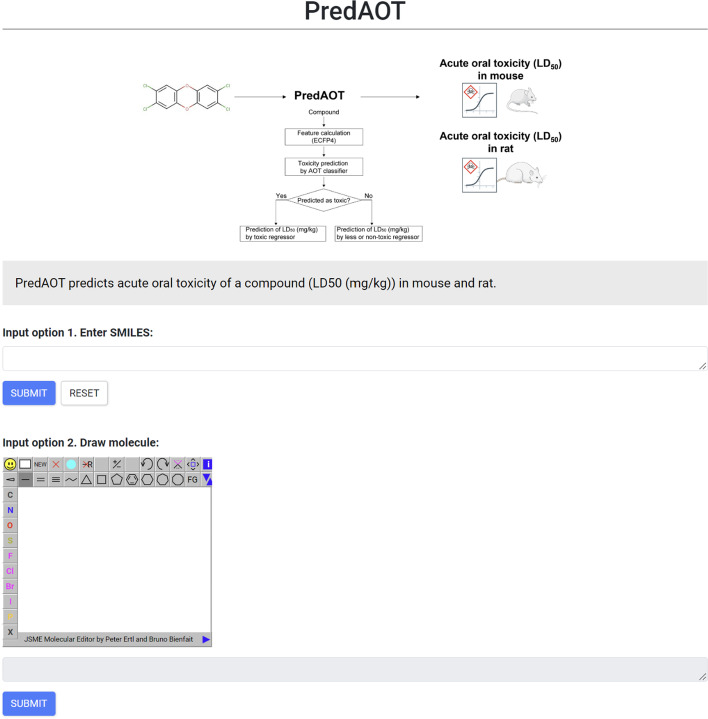
User interface of the PredAOT web server

In the present study, we developed a computational framework called PredAOT, which predicts the AOT of a given compound in mice and rats. PredAOT first classifies the given compound as “toxic” or “less or non-toxic”, and then further qualitatively predicts the LD_50_ value using a regression model. PredAOT is trained with information on the AOT of 6,226 and 6,238 compounds in mice and rats, respectively. PredAOT has the advantage of predicting AOT in mice and rats simultaneously, and its prediction performance is similar to or better than that of existing tools. The web server for implementing PredAOT is available at https://predaot.netlify.app/ (Fig. 6). PredAOT will be a useful tool for the quick and accurate prediction of the AOT of small compounds in mice and rats for successful drug development.

## Methods

### Data preparation

Information on acute oral toxicity (AOT) of 6,226 compounds determined in mice was obtained from the OCHEM database [7], and information on AOT of 6,238 compounds determined in rats was obtained from the literature [6]. We defined compounds with lethal dose 50 (LD_50_) values ≤ 300 mg/kg as “toxic” and compounds with LD_50_ values > 300 mg/kg as “less or non-toxic”. The dataset was divided into training (80%) and test (20%) datasets. The training dataset was used for hyperparameter optimization, and the test dataset was used for model evaluation (Fig. 1). During the model training and evaluation, we used log10 transformed LD50 values.

### Preparation of molecular features

The structures of the compounds were presented in the simplified molecular-input line-entry system (SMILES) format [14]. To train message-passing neural networks (MPNNs), we used the Chemprop Python package [15]. The RDKit Python package was used to calculate the molecular fingerprint. Extended connectivity fingerprints with a maximum diameter parameter of 4 (ECFP4) were used [16].

### Optimization of machine learning algorithms

In this study, we tested six different machine learning (ML) algorithms, including MPNN, MPNN with molecular fingerprints, MPNN with molecular descriptors, random forest (RF), support vector machine (SVM), and artificial neural network (ANN) models, to build both classification and regression models for predicting AOT in mice and rats. Here, the RF, SVM, and ANN models were trained using molecular fingerprints as input features. MPNN learns directly from a molecular graph to predict molecular properties [15]. ANN is an ML algorithm inspired by the biological neuronal network of the human brain [17]. The ANN structure consisted of an input layer, hidden layer(s), and an output layer. The ANN learns non-linear relationships from the data. RF is an ensemble learning algorithm that constructs multiple decision trees [18]. The ANN algorithm was implemented using the Keras package (version 2.2.5) with TensorFlow backend (version 2.0.0) [19]. The RF and SVM algorithms were implemented using the *scikit-learn* Python package [20].

To build the optimal model with the best prediction performance, hyperparameter optimization was performed. For the classification model, we selected the optimal hyperparameter that showed the highest accuracy (ACC) using the grid-search cross-validation method. In addition, for the regression model, we selected the optimal hyperparameter that showed the lowest root mean square error (RMSE) using the grid search cross-validation method.

Five metrics were used to evaluate the performance of the classification model: ACC, area under the receiver operating characteristic (AUROC), Matthew’s correlation coefficient (MCC), positive predictive value (PPV), and negative predictive value (NPV). In addition, to evaluate the regression model performance, two performance metrics were used: the RMSE and R-squared value (R^2^).

## Data Availability

The data and source code are available at https://github.com/CSB-L/PredAOT, and the PredAOT web server is available at https://predaot.netlify.app. The datasets supporting the conclusions of this study are available from the corresponding author upon reasonable request.

## Abbreviations

ACC: Highest accuracy
ANN: Artificial neural network
AOT: Acute oral toxicity
AUROC: Area under the receiver operating characteristic
LD_50_: Lethal dose 50
ML: Machine learning
MCC: Matthew’s correlation coefficient
MPNN: Message passing neural network
NPV: Negative predictive value
PPV: Positive predictive value
RF: Random forest
RMSE: Root-mean-square error
SMOTE: Synthetic minority oversampling technique
SMILES: Simplified molecular-input line-entry system
SVM: Support vector machine
